# Putting self‐regulated learning in context: Integrating self‐, co‐, and socially shared regulation of learning

**DOI:** 10.1111/medu.14566

**Published:** 2021-06-07

**Authors:** Derk Bransen, Marjan J. B. Govaerts, Ernesto Panadero, Dominique M. A. Sluijsmans, Erik W. Driessen

**Affiliations:** ^1^ School of Health Professions Education (SHE) Maastricht University Maastricht The Netherlands; ^2^ Department of Educational Development and Research Faculty of Health, Medicine and Life Science Maastricht University Maastricht The Netherlands; ^3^ Facultad de Psicología y Educación Universidad de Deusto Bilbao España; ^4^ IKERBASQUE Basque Foundation for Science Bilbao Spain; ^5^ Faculty of Social Sciences Radboud University Nijmegen The Netherlands

## Abstract

Processes involved in the regulation of learning have been researched for decades, because of its impact on academic and workplace performance. In fact, self‐regulated learning is the focus of countless studies in health professions education and higher education in general. While we will always need competent individuals who are able to regulate their own learning, developments in healthcare require a shift from a focus on the individual to the collective: collaboration within and between healthcare teams is at the heart of high‐quality patient care. Concepts of collaborative learning and collective competence challenge commonly held conceptualisations of regulatory learning and call for a focus on the social embeddedness of regulatory learning and processes regulating the learning of the collective. Therefore, this article questions the alignment of current conceptualisations of regulation of learning with demands for collaboration in current healthcare. We explore different conceptualisations of regulation of learning (self‐, co‐, and socially shared regulation of learning), and elaborate on how the integration of these conceptualisations adds to our understanding of regulatory learning in healthcare settings. Building on these insights, we furthermore suggest ways forward for research and educational practice.

## Abstract 2

Bransen et al. explore the concept of 'self' to argue that the health professions need to shift from striving to optimize self‐regulation to understanding how to effectively regulate across self‐, co‐, and shared‐regulation.

## 1 INTRODUCTION AND PROBLEM STATEMENT

The necessity for healthcare professionals to regulate their learning is widely acknowledged due to positive associations with high‐quality healthcare and lifelong learning.^1^, ^2^ For example, safeguarding high standards in healthcare requires physicians to monitor relevant developments in continuously and rapidly changing healthcare practices and to align appropriate learning opportunities with personal learning needs and learning goals.^3^ Hence, physicians have to engage in self‐regulated learning (SRL) to develop and maintain competence.^4^ Generally, self‐regulated learners are considered to be “meta‐cognitively, motivationally, and behaviourally active participants in their own learning”.^5^ In pursuit of and committed to their goals, they design and implement strategies aligned with these goals, monitor progression towards these goals, followed by reflection and – when applicable – formulation of new learning goals.^6^, ^7^

Because of its relevance for education and practice, and as SRL skills can be learnt and therefore taught, health professions curricula build in elements to support and facilitate the development of students’ SRL.^8^, ^9^, ^10^ For example, many curricula implement portfolio systems that stimulate or require students to reflect on progress and formulate personal learning plans,^11^ or include problem‐based learning principles to stimulate students’ self‐directedness.^12^, ^13^ Research findings consistently show that the ability to regulate one's learning and professional development is associated with positive outcomes.^14^ For example, SRL has been related positively to medical students’ clinical skill performance,^15^, ^16^ their overall academic achievement,^17^ and student well‐being.^18^ However, we argue that there is a need and responsibility for health professions education and research to look beyond the self in order to adequately prepare students for practice, and to help professionals maintain and develop competence.

Health professions education (and healthcare practice, for that matter) has traditionally been characterised by a focus on the individual; education focuses on individual learners whom we licence individually after extensive individual assessment, and whom we teach to regulate their learning processes and activities on an individual level.^19^ One might argue that health professions education aims to move beyond the individual by including competency domains such as “Collaboration” in curriculum and assessment frameworks.^20^ However, while described as ‘*effectively working within a healthcare team to achieve optimal patient care*’, learners’ proficiency as collaborators is still primarily based on their individual performance, even when evaluated in collaborative situations.^21^ Notwithstanding, present‐day healthcare is increasingly team‐based, delivered by healthcare teams, often consisting of healthcare professionals collaborating across specialities and professions.^22^, ^23^ As the main purpose of health professions education is to prepare students for this collaborative practice, it is essential that conceptualisations of regulation of learning align with the organisation and demands of learning and working in healthcare teams.

Geared to the growing reliance on healthcare teams for high‐quality healthcare delivery, health professions education research has started to explore the concept of collective competence.^22^, ^23^, ^24^ The essence of collective competence is that the whole can be more (or less, for that matter) than the sum of its parts, and relates to the ‘dynamic, context‐dependent, distributed capacity of a team, which is difficult to trace back to any one individual team member’.^25^ In other words, ‘*teams can be competent when one team member is incompetent, and competent individuals can form an incompetent team’*.^19^ Although ensuring an individual physician's competence is and remains essential, providing high‐quality healthcare thus requires assurance of the healthcare team's collective competence. To maintain and develop collective competence, it is essential that healthcare teams are able to engage in ongoing collaborative learning. Collaborative learning refers to learning that occurs when team members who have a collective goal interact about features of their shared tasks in order to attain their goals and by means of which they develop a set of integrated practices.^26^, ^27^ As such, collaborative learning stretches beyond the individual and emphasises the interdependence among team members.^28^ Collaborative learning may, for example, occur during trauma teams’ evaluation of healthcare delivery, when surgical teams start implementing new technology, or when students collaborate in performing learning tasks. Whenever collaborative learning is considered essential, the team's ability to regulate their learning becomes of equal importance. In other words, if we agree that high‐quality care hinges upon collaborative learning in healthcare teams, we should also focus on how to foster effective regulation of learning in order to develop and maintain collective competence. However, questions can be raised about the extent to which conceptualisations of regulation of learning in health professions education and research kept pace with the demands for collaborative learning and competence in healthcare practice.

## 2 CONCEPTUALISATIONS OF REGULATION OF LEARNING

### 2.1 Self‐regulated learning: Focus on the individual

Chronologically, the first conceptualisations in the regulation of learning theory focussed on the *self*, that is, on how an individual student or professional regulates his or her individual learning. Some of the earliest attempts to conceptualise the regulation of learning were made in the late 1980s by Zimmerman^5^, ^29^ and by Boekaerts,^30^, ^31^ with their SRL models having been adapted, expanded, and used for further research ever since.^10^ The first SRL models labelled processes *within* the individual – varying in their emphasis on either (meta)cognitive, motivational, or emotional aspects – as modus operandi of regulation of learning. Consequently, researchers interested in *self‐*regulation of learning focussed on processes within the individual as the unit of analysis.^32^ Likewise, the majority of research (both within and outside the context of healthcare education) into SRL is conducted through collecting self‐reported data.^33^ Research on regulation of learning within healthcare (educational) settings with a strong focus on the individual is reflected in studies focussing on sub‐components of SRL such as individualised learning plans,^34^, ^35^, ^36^, ^37^ and self‐monitoring of performance.^38^, ^39^, ^40^

### 2.2 Co‐regulated learning: Focus on interaction between individual and context

While early conceptualisations of regulation of learning (ie *self*‐regulation of learning) emphasise processes within the individual learner, the term co‐regulated learning (CRL) was coined in the late 1990s to capture the social and contextual influences on the regulation of learning.^41^, ^42^, ^43^ The concept of CRL emerged from sociocultural learning theories that focus on how learners’ cognitions, emotions, and motivation for learning are mediated through social interactions with others in the environment.^41^ CRL thus builds on the notion that we need to go beyond regulatory processes within the individual in order to describe the regulation of learning satisfactorily, and the unit of analysis in CRL always is the interaction between the individual and (others in) the context.^32^ More specifically, CRL refers to non‐reciprocal engagement in regulatory processes and activities, with the ‘co‐regulator’ guiding the regulation of the ‘co‐regulated’. Conceptually, CRL is therefore considered an ‘unevenly distributed’ form of social regulation, in that a single or multiple group member(s) regulate(s) the learning activities of other individuals in the group.^44^ Essential to CRL are social interactions between learners or professionals through which their learning processes, including processes relevant for the regulation of their learning, are mediated.^45^, ^46^ Thus, through engaging in others’ regulatory activities – such as goal setting, performance monitoring, and reflection – the ‘co‐regulator’ mediates (ie co‐regulates) the metacognitive and cognitive activities of the ‘co‐regulated’, thereby influencing the regulation of his or her learning processes.^41^, ^45^ Students or professionals can trigger CRL by summarising, requesting information, or giving explanations,^47^ or through paraphrasing, requesting judgements of learning, giving prompts for thinking and reflection.^41^ Box 1 provides an example.

BOX 1 **The co‐regulation of learning how to close a wound after surgery**

A student formulated a learning goal aimed at mastering basic techniques of wound closure. The supervising surgeon provides the student with the opportunity to pursue this goal by allowing the student to start the procedure of closing the wound. The role of the supervising surgeon consists of actively participating in the student's regulation of learning (ie co‐regulation). Before the student starts, the surgeon may ask about the steps the student intends to take to close the wound successfully (co‐regulation of strategic planning). Similarly, when the student is actively closing the wound, the surgeon may ask if the student is on the right track thus far (ie co‐regulation of monitoring). After the student finishes closing the wound, the surgeon may ask about potential difficulties the student may have experienced and how he or she may improve future efforts (co‐regulation of reflection and adaptation). After co‐regulatory interactions with her supervisor, the student may then actively engage in and reflect on learning activities with the aim of transferring relevant skills for regulation of learning to other, unsupervised, learning tasks. Through engaging in the students’ regulation of learning, the supervising surgeon helps to support the application and development of self‐regulatory learning skills.

Importantly, CRL can take different forms, depending on the learning task, setting and/or relationships between co‐regulator and learner. For example, power dynamics in hierarchical relationships or (perceived) differences in the level of expertise may influence the nature – and potentially effectiveness – of CRL. Co‐regulation by peers may therefore differ substantially from supervisors’ CRL in terms of goals and outcomes. Within health professions education, emergent research on co‐regulation of learning is providing insight into the different manifestations and foci of CRL engagement. Research findings suggest that medical students differ in whom they engage as well as the purpose of engaging others’ CRL. For instance, novice students seem to favour peers to discuss their learning goals, whereas experienced students favour more experienced healthcare professionals to reflect on professional identity formation.^48^ Other studies into CRL adopted a social network perspective, and examined characteristics of the networks students’ deploy when regulating their learning. Findings revealed that, in particular, the interaction frequency with which others are engaged in CRL positively relates to students’ self‐reported regulation of learning proficiency.^49^

### 2.3 Socially shared regulation of learning: Focus on the team within context

At the start of the 21st century, fuelled by the increasing importance and need for collaborative learning, research started exploring how groups regulate their collective learning and performance in a distributed fashion. The term socially shared regulation of learning (SSRL) was coined to explain such regulatory actions. Generally, SSRL describes how teams regulate their collaborative learning and emphasises interdependency among members of a group or team. SSRL focuses on processes through which team members share the regulation of their collective learning activities, directed towards the pursuit of their jointly constructed goals.^41^, ^50^ Similar to CRL, SSRL reflects a mode of regulatory learning in which the regulation is shared between individuals. The main difference, however, is that CRL involves one (or more) group members to guide the regulation of an individual learner (making it an ‘unevenly distributed’ form of social regulation), whereas SSRL is characterised by group members’ reciprocal engagement in regulatory activities and processes. SSRL is therefore considered an ‘evenly distributed’ form of social regulation in which the regulation is shaped by and arises through the interactions between members of the group.^44^ Therefore, the units of analysis in SSRL are the collective, the system, as well as the individual within the system.^32^, ^51^

Collaborative learning in medical practice may be prone to challenges. For example, fluid healthcare teams in which team members reshuffle constantly, time constraints, or hierarchy within healthcare teams may influence the extent to which collaborative learning is actually taking place. Collaborative regulation of such learning may subsequently be even more difficult. Hadwin, Järvelä, and Miller^52^ were among the first to describe a theoretical model SSRL. Their conceptualisation of collaborative regulation of learning includes four phases that jointly describe the modus operandi of SSRL.^52^ Notably, these phases roughly correspond to the four phases in Winne and Hadwin's SRL model.^53^ In phase one, teams engage in the co‐construction and negotiation of a shared understanding or perception of the (learning) task at hand. In phase two, teams co‐construct shared goals to effectively complete the task and design a plan for how to tackle the task collectively. In phase three, the team monitors their progression towards the goal, to which collaboration is strategically coordinated. Perceptions and understanding of the task, their goal(s), strategies, or plans might be adjusted based on their collective monitoring of goal progression. Lastly, in phase four, teams evaluate the process, which might provide input for adaptation of future regulation, learning and performance. Drawing on Edmondson's work about how introducing new technology influences teamwork, Box 2 provides an example of how healthcare teams may engage in socially shared regulation of learning.^54^ We acknowledge that the example in Box 2 may not reflect common, day‐to‐day practice. However, the research the example is based on, lends itself well to explain how SSRL may occur in clinical workplaces. Although SSRL may be particularly relevant in cases of disruptive events that force healthcare teams to re‐direct their learning endeavours (such as in the example in Box 2), SSRL is not exclusive to such events. Other examples in health professions education and practice that may appeal to teams’ engagement in SSRL or components of SSRL include evaluation of corporate training systems, the building of collaborative communities of practice,^55^ team reflection, or medical students’ collaborating in co‐constructing a shared perception of their learning tasks.

It is important to note that describing SSRL in terms of distinct phases refers to a theoretically ideal situation. In practice – especially in the unpredictable and dynamic context of healthcare practice – teams might not go through the phases in the abovementioned order, or might merge phases (as in the example in Box 2). However, research suggests that teams that go through these phases tend to be more successful in learning in and adapting to new situations.^54^ Healthcare education research into SSRL is limited, although seemingly similar concepts emerged from research on team learning (eg team reflexivity).^56^, ^57^, ^58^ Given that healthcare quality is associated with the quality of learning and working in healthcare teams,^19^, ^59^ the conceptualisation of SSRL provides a valuable lens through which we might be better able to examine and understand how regulatory processes support collaborative learning.

BOX 2 **SSRL when adopting new technologies**

Adopting new technology may raise challenges to healthcare teams, as habitual routines may be disrupted. Teams then have to go through a learning process, which involves creating a shared willingness to start using the technology (motivation) as well as a shared mental model of what the new technology implies, not just in terms of new knowledge and skills but also in terms of potentially changing tasks and responsibilities of team members (SSRL phase 1). In her paper about implementation of a minimally invasive cardiac surgery innovation, Edmondson described how surgical teams engaged in learning to implement this technology. Her findings showed that teams who were effective in adapting to a new reality, spent time on engaging all team members in the team effort (both intellectually and emotionally) as well as creating a clear definition of the team's goals, roles, and responsibilities in the implementation process (SSRL phases 1 and 2). Successful OR teams then proceeded by jointly developing strategies for learning such as trial sessions and ongoing monitoring of the implementation process (SSRL phases 2 and 3). Then, OR teams continually engaged in monitoring of and reflection on their progress, through processes of attempting new behaviours and debriefing (reflection and debriefing) in order to learn (SSRL phase 4).

## 3 INTEGRATING SELF‐, CO‐, AND SOCIALLY SHARED REGULATION OF LEARNING IN EDUCATION AND HEALTHCARE

During collaborative learning situations, teams and team members may engage in self‐, co‐, as well as socially shared regulation of learning. The balance within collaborating teams regarding their engagement in regulatory processes and activities may shift across individuals and over time, based on characteristics of individual team members, the team composition and relationships between team members, social connectedness, as well as features of task and context. This shifting balance may subsequently result in varying levels of SRL, CRL or SSRL, depending on whether regulatory engagement is evenly (SSRL) or unevenly (CRL) distributed across team members. See Box 3 for an example.

To function productively as a collective, individual SRL geared towards collective goals is crucial.^52^ When teams engage in collaborative learning, individual team members will therefore engage in self‐regulating their own learning processes and activities; even during collaborative learning, individual team members will activate strategies individually and monitor and regulate their individual efforts.^60^ Team‐level CRL may emerge during collaborative learning in cases when an individual team member takes control of or stimulates another team member's regulation processes or activities.^60^ As such, CRL can play a mediational or transitional role towards productive self‐regulation, yet also shared regulation of learning, depending on whether co‐regulation is geared towards an individual team member's regulation (SRL) or the regulation of team as a collective (SSRL).^52^ The team member in Box 3 who expresses concerns about whether all of their collective goals are adequately evaluated, for example, serves as a co‐regulatory mechanism through which the agency of regulation of learning shifts towards the collective. SSRL during collaboration may emerge when all team members regulate learning processes collectively, such as co‐constructing goals or task perceptions. When teams engage in SSRL, team members collectively take metacognitive control of the team's tasks by means of adjusting behaviours, cognitions, and motivations, based on requirements for completion of their tasks.^52^

BOX 3 **Integrating self‐, co‐, and socially shared regulation of learning**

To elucidate the integration of different levels of regulation of learning during collaborative learning, returning to the implementation of new technology in Box 2 might be helpful. Each individual team member of the surgical team activates individual regulatory processes such as effort regulation, individual monitoring of the task and his or her performance (SRL). Through negotiations and discussions, the team members co‐construct a shared perception of the task as a team, and collectively formulate goals and strategies to accomplish the task (SSRL). During evaluation after the first attempts of using the new technology, one team member may notice that another team member is not picking up essential skills and helps him to adopt another learning strategy and to better monitor his performance throughout the procedure. (CRL aimed at other's SRL). Similarly, one team member may notice that the team is overlooking evaluating one of their collectively set goals and draws the team's attention to this goal (CRL aimed at the team's SSRL). As such, the team is able to regulate their collaborative learning efforts through concurrent engagement in SRL, CRL and SSRL.

In any collaborative learning, engagement in momentary co‐regulatory interactions may occur within episodes of both SSRL and SRL. Thus, learners may concurrently engage in different forms of regulation. The three levels of regulatory learning (SRL, CRL, and SSRL) may therefore best be considered as embedded in one another during collaborative learning situations.^41^, ^44^ During collaborative learning, teams may not always engage in either CRL or SSRL (or SRL for that matter). Whether a team will engage in either CRL or SSRL (or both) is context‐ and situation‐specific. For example, if the team leader (Box 3) is highly directive, fully guiding the regulation of learning (ie CRL), learning will likely be regulated without engagement in SSRL. Therefore, whether CRL is transitional towards other modes of regulation depends on dynamics within the team and team leadership as well as requirements of the learning task.

## 4 A WAY FORWARD

### 4.1 Implications for research

Importantly, conceptualisations of regulation of learning in the present article refer to an idealised and theoretical situation, which may differ from actual work settings. However, such models may provide useful frameworks for future research to disentangle how regulation of learning may occur in collaborative settings. While various levels of regulatory learning are increasingly explored in health professions education research,^45^, ^48^, ^61^, ^62^, ^63^ studies predominantly focus on processes within the individual or the individual in interaction and less on how teams regulate their collaborative learning. Given the demands for collaboration in current healthcare, it is important to widen our views of regulatory learning, and we propose that future research adopts a multi‐level and integrated perspective, focussing on the levels of self‐, co‐, as well as socially shared regulation of learning in healthcare (education) settings.

This first and foremost implies that researchers interested in the regulation of learning should add SSRL to the equation that is currently dominated by SRL, and to a lesser extent, by CRL. The importance of focussing on social regulation to understand collaborative learning has recently been underlined in the context of health professions education.^64^ Building on related concepts, such as team reflexivity,^57^, ^58^ health professions education research could shift attention to team‐level regulatory processes and activities, aiming to understand how teams – as well as individual team members – shape their regulation towards their collective goals. Furthermore, to improve our understanding of the regulation of learning, future studies could aim to disentangle the interrelatedness of SRL, CRL, and SSRL during collaborative learning. Specifically, researchers may want to explore the mediating role of CRL towards productive SRL and SSRL, and how regulatory interactions affect learning and performance. Because CRL can provide the affordances and constrains for other modes of regulation, a thorough understanding of the mechanisms by which it may exert its influence is essential.

Much of the SRL data in health professions education research (and CRL data for that matter) is collected through subjective self‐reports,^33^ exploring participants’ perceptions of their regulatory activities. However, these perceptions often differ from their actual behaviour.^65^ To overcome these limitations, recent trends draw on technological advancements and point to collecting multimodal data.^52^, ^66^ This involves collecting data from different data channels (ie modalities),^52^ for example objective physiological and subjective self‐report data, allowing researchers to examine features and phases of regulatory learning in complex collaborative learning situations.^67^ Through collecting objective data, we are able to make visible what otherwise remains invisible, such as effort regulation, increased attention, and confusion that may take place during episodes of SRL, CRL, and/or SSRL. For example, recent studies use data sources such as 360‐degree cameras and electro‐dermal measures to examine group members’ shared monitoring of collaborative learning,^68^ or collect physiological data such as heart rate and skin conductance measures (eg to measure emotional reactions) during collaborative learning situations.^66^ Triangulating data from different sources (both objective and subjective data), may help us to better describe levels and outcomes of regulation of learning in various settings. To improve our understanding of the regulation of learning during collaboration, we can draw on simulation‐based research, in particular,^69^ as this more easily allows incorporation of technology. Simulation‐based research settings seem therefore eminently suitable for helping scholars analyse and disentangle complex phenomena that are difficult to uncover,^70^ such as regulatory learning processes.

Ethnographic research might offer unique and new opportunities to further our understanding of regulatory learning processes. Direct observation of healthcare teams, either in real‐life settings or in simulation settings, may enable exploration of regulatory behaviour as it occurs during the performance of authentic tasks and how different regulatory forms may be embedded in one another. Additionally, observing regulatory behaviour allows for examination of the distinction of unevenly distributed CRL and evenly distributed SSRL. This distinction is theoretical and conceptual and may reflect theoretically ideal regulatory patterns. Investigating the extent to which regulation of learning is distributed across team members within clinical settings may help describe and improve real‐world practices.

### 4.2 Implications for health professions education

When collaborative learning is considered important for healthcare professionals, regulation of collaborative learning becomes equally important. Therefore, elements that support, stimulate, and facilitate the regulation of collaborative learning should permeate healthcare professions curricula. First and foremost, increasing awareness of different regulatory levels is vital. Currently, most healthcare professions curricula seem to pay more attention to SRL than to CRL and SSRL. Increasing team members’ awareness of each other's knowledge, activities, emotions, motivation, and views of the group's functioning as a collective is a crucial starting point to support development of CRL and SSRL.^50^ To help make explicit what often remains implicit, discussions that focus on team members’ awareness of own and other regulatory learning processes could be stimulated during debriefing sessions of simulation‐based team training sessions, for example.^71^, ^72^

An important implication is that health professions education programmes create a learning environment that fosters the development of individual as well as collective regulatory competence. If one of the aims of healthcare professions education is to promote collaborative learning, curricula must include learning tasks that require collaborative learning as well as regulation of that learning. These learning tasks should provide students with information that is relevant for developing such skills. It is then crucial that attention is paid to the provision of feedback that is explicitly aimed at specific self‐, co‐, and shared regulatory learning processes and activities.^73^

## 5 CONCLUSION

Learning – and therefore regulation of learning – within the health professions domain takes place at different levels, with different levels of regulation of learning being embedded in one another. While the importance of collaboration and collective competence for healthcare professionals is increasingly recognised, attention to how healthcare teams regulate their collaborative learning has yet to gain momentum. We, therefore, may want to shift from an exclusive focus on how to optimise *self*‐regulation of learning, to the broader perspective of how to most effectively regulate learning, depending on the level at which it takes place. Truly unravelling regulation of learning within the healthcare domain therefore means unravelling the levels of self‐, co‐, and socially shared regulation of learning. Only then are we able to help future healthcare professionals to develop the skills that are necessary to function productively within the complex, unpredictable, and collaborative context of healthcare delivery.

## AUTHOR CONTRIBUTIONS

DB is the principle author of the work. All authors contributed to the conception and/or refinement of the work. DB drafted the initial manuscript. All authors contributed to revisions of the paper. All authors approved the final manuscript for publication.
